# Deep Brain Stimulation in Treatment-Resistant Depression: A Systematic Review and Meta-Analysis on Efficacy and Safety

**DOI:** 10.3389/fnins.2021.655412

**Published:** 2021-04-01

**Authors:** Youliang Wu, Jiajie Mo, Lisen Sui, Jianguo Zhang, Wenhan Hu, Chao Zhang, Yao Wang, Chang Liu, Baotian Zhao, Xiu Wang, Kai Zhang, Xuemin Xie

**Affiliations:** ^1^Department of Functional Neurosurgery, The Second Affiliated Hospital of Guangzhou University of Chinese Medicine, Guangzhou, China; ^2^Department of Neurosurgery, Beijing Tiantan Hospital, Capital Medical University, Beijing, China; ^3^Department of Neurosurgery, Beijing Neurosurgical Institute, Capital Medical University, Beijing, China

**Keywords:** deep brain stimulation, major depressive disorder, nerve nuclei, treatment-resistant depression, psychiatric surgery

## Abstract

**Objective:** Deep brain stimulation (DBS) has shown promising outcomes as new therapeutic opportunities for patients with treatment-resistant depression (TRD) who do not respond adequately to several consecutive treatments. This study aims to systematically review and conduct a meta-analysis on the efficacy and safety of DBS for TRD.

**Method:** The literature was comprehensively reviewed using Medline, Google scholar, Cochrane library, Embase, and World Health Organization International Clinical Trials Registry Platform until January 2019. The studied outcomes included response, remission, recurrence, and adverse events (AEs) rates, and were reported as the rate ratio (RR) or pooled estimate with a 95% confidence interval (95% CI). Heterogeneity was measured by an I-square test and a sensitive analysis.

**Results:** A total of 17 studies involving 7 DBS targets were included. For efficacy, DBS treatment was statistically beneficial for TRD, and the response, remission, and recurrence rates were 56% (ranging from 43 to 69%), 35% (ranging from 27 to 44%), and 14% (ranging from 4 to 25%), respectively. However, only two randomized-controlled trials (RCTs) considered the invalidity of DBS (*RR* = 1.45, 95% CI = 0.50–4.21). For safety, the AEs rate was 67% (ranging from 54 to 80%). The AEs were common and moderate, but the problems related to suicide and suicidal ideation should not be underestimated.

**Conclusion:** These findings suggest that DBS for TRD is considered promising, which should be confirmed by well-designed and large sample studies. Future basic research and comprehensive clinical trials are needed to reach better understanding on the mechanisms of action and optimal targeted structure.

## Highlights

- DBS is considered as an effective treatment for TRD.
- The AEs induced by DBS were common and moderate, but the problems related to suicide and suicidal ideation should not be underestimated.
- Future basic research and comprehensive clinical trials are needed to better understand the mechanisms of action and optimal modulatory structure.

## Introduction

Depression is the most common mental disorder and a leading cause of disability worldwide. From 1990 to 2017 the number of incident cases of depression worldwide increased by 49.86% with 25,8 million in 2017 (James et al., 2018). Depression is characterized by a depressed mood, decreased energy, psychomotor change, reduced concentration, indecisiveness, decreased self-esteem, guilt, suicidal ideation, decreased interest, and nutritional and weight changes (Prichep and John, 1992; Hastings et al., 2004; Nubukpo et al., 2004; Mohammadi et al., 2018). Depression is the leading cause of disability worldwide as measured by years lived with disability (Biesheuvel-Leliefeld et al., 2015). There are several psychopharmacological treatments for depression; however, a third portion of the patients do not respond adequately to the psychological and pharmacological treatments. Failure to respond to one or more adequate antidepressant treatments is defined as the presence of treatment-resistant depression (TRD). Patients with TRD cannot be cured quickly (Vieta and Colom, 2011), and 20–80% of patients encounter relapse within 5 years, in spite of maintenance therapy (Nierenberg, 2001; Schlaepfer and Bewernick, 2015). Several non-pharmacological modalities have been developed for treatment of patients with TRD such as vagus nerve stimulation, electroconvulsive therapy (ECT), epidural cortical stimulation, repetitive transcranial magnetic stimulation, transcranial direct current stimulation, and deep brain stimulation (DBS) and some of them have shown promising outcomes (Bystritsky et al., 2011; Kuo et al., 2014; Pal et al., 2015; Yadollahpour et al., 2016, 2017; Brennan et al., 2017). DBS is a surgical procedure in which the stereotactically implanted electrode delivers continuous electrical stimulation into specific neuroanatomical targets leading to therapeutic effects in different disorders (Pallanti, 2008; Bergfeld et al., 2016). DBS has been established as a therapy for Parkinson's disease (PD), essential tremor and movement disorders; however, its effectiveness for the management of treatment-resistant depression (TRD) remains unclear. Currently, patients with TRD who do not respond adequately to several consecutive antidepressive treatments are increasingly asking about deep brain stimulation (DBS) as an option (Torres, 2008; Taghva et al., 2013). DBS is a therapeutic technique in the early phase of evaluation (level III) (Schlaepfer and Bewernick, 2015). However, its practical function remains controversial. Since 2005, over 200 patients diagnosed with TRD received experimental DBS. Several DBS targets have been used for TRD, including, subcallosal cingulate gyrus (SCG), ventral capsule/ventral striatum (VC/VS) or anterior limb of internal capsule (ALIC), nucleus accumbens (NAc), epidural prefrontal cortical (EpC), ventral anterior limb of the internal capsule (vALIC), medial forebrain bundle (MFB), lateral habenula (LHb), inferior thalamic peduncle, supero-lateral branch of the medial forebrain bundle (sMFB), and the posterior gyrus rectus. Moreover, the lateral habenula and inferior thalamic nucleus are also described in published case reports (Jiménez et al., 2005; Sartorius et al., 2010). Previous open-label trials (Mayberg et al., 2005; Lozano et al., 2008, 2012; Malone et al., 2009; Bewernick et al., 2010; Nahas et al., 2010; Kennedy et al., 2011; Holtzheimer et al., 2012; Puigdemont et al., 2012, 2015; Merkl et al., 2013; Schlaepfer et al., 2013; Dougherty et al., 2015; Accolla et al., 2016; Bergfeld et al., 2016; Fenoy et al., 2016; Williams et al., 2016) support that DBS, as a treatment, was conducive to TRD, but two randomized-controlled trials (RCTs) found conflicting results concerning placebo effects (Dougherty et al., 2015; Puigdemont et al., 2015). The results of an RCT trial did not demonstrate a significant difference in response rate between the DBS-active and shame-controlled group (20 vs. 14.3%) (Dougherty et al., 2015). This discrepancy potentially results from an overestimation of the efficacy associated with former open-label trials. Moreover, the difference in the sample size, patients' features, and the DBS parameters could be the sources of the discrepancy. The rate or ratio, widely used in clinical practice, is appropriate to describe the frequency of the disease distribution, the overall effect of the intervention and the accuracy of the diagnosis and prognosis of the disease. The promising features of the rate parameter are intuitiveness and comparableness, but the uneven quality of various studies makes the results different. Therefore, a meta-analysis of rate is expected to quantitatively combine the pooled effect among multiple studies and to obtain more reliable conclusions. Lacking well-designed RCTs and large samples renders a meta-analysis difficult.

Therefore, the present study was aimed to systematically review and conduct a meta-analysis to evaluate the efficacy and safety of therapeutic modalities, specifically DBS for treatment of TRD.

## Materials and Methods

This meta-analysis was conducted in accordance with the Preferred Reporting Items for Systematic Reviews and Meta-Analyses (PRISMA) (Moher et al., 2009) and Cochrane guidelines (Higgins, 2008).

### Eligibility Criteria

The inclusion criteria of this study, according to the PICOS checklist (Higgins, 2008), are as follows: (1) Population: patients diagnosed with TRD based on the diagnostic and statistical manual of mental disorders, 4th edition (DSM-IV), and treatment resistance was defined as a failure of antidepressant therapies at an adequate dosage and duration; (2) Intervention: DBS targeting various nerve nuclei; (3) Control: placebo or sham stimulation in the trials if the data was available; (4) Outcomes: response decline, remission and recurrence rates, referred to as “efficacy,” and adverse events (AEs) rate, referred to as “risk” and (5) Study design: RTCs and open-label studies were included.

### Information Sources and Data Collection

Two independent authors (JM and WH) constructed the corresponding search strategies in the Medline, Google scholar, Cochrane library databases, Embase and World Health Organization International Clinical Trials Registry Platform up to January 2019. The search results from the databases were obtained using the terms “deep brain stimulation” OR “DBS” AND “depression” OR “depressive disorders.” We searched for eligible studies by scanning the abstracts from the original articles and screening the list of references for relevant publications. Additionally, other references were manually scanned from relevant reference reports and clinical trial websites. Only eligible studies that met the predefined criteria were input into a bibliography management system.

### Data Items

For accuracy and completeness, two reviewers (JM and KZ) independently checked the full articles and extracted the information corresponding to the characteristics of the study population, the details of the surgery, the outcome measures, and the adverse events. We recorded the data, including the Hamilton Rating Scale for Depression (HRSD), Montgomery-Asberg Depression Rating Scale (MADRS), adverse events, suicidal ideation and suicide, in each target location. Discrepancies were resolved by discussion and consensus. Cochrane tools were not used to assess the quality of the included studies, because most were open-label studies.

### Summary Measures

A meta-analysis was conducted to calculate the studies outcomes, including the response, remission, recurrence and adverse events rate, using Stata software (version 13.0). A 50% or greater improvement from the baseline score to end of treatment on a depression rating scale (for example, the HDRS or the MADRS) was defined as the clinical response. A clinical remission was defined as a score on a depression rating scale within the normal range (for example, HDRS of 7 or less or MADRS of 12 or less). The adverse effects were evaluated by examining the proportion of adverse events.

## Results

### Study Selection and Study Characteristics

**Figure 1:**
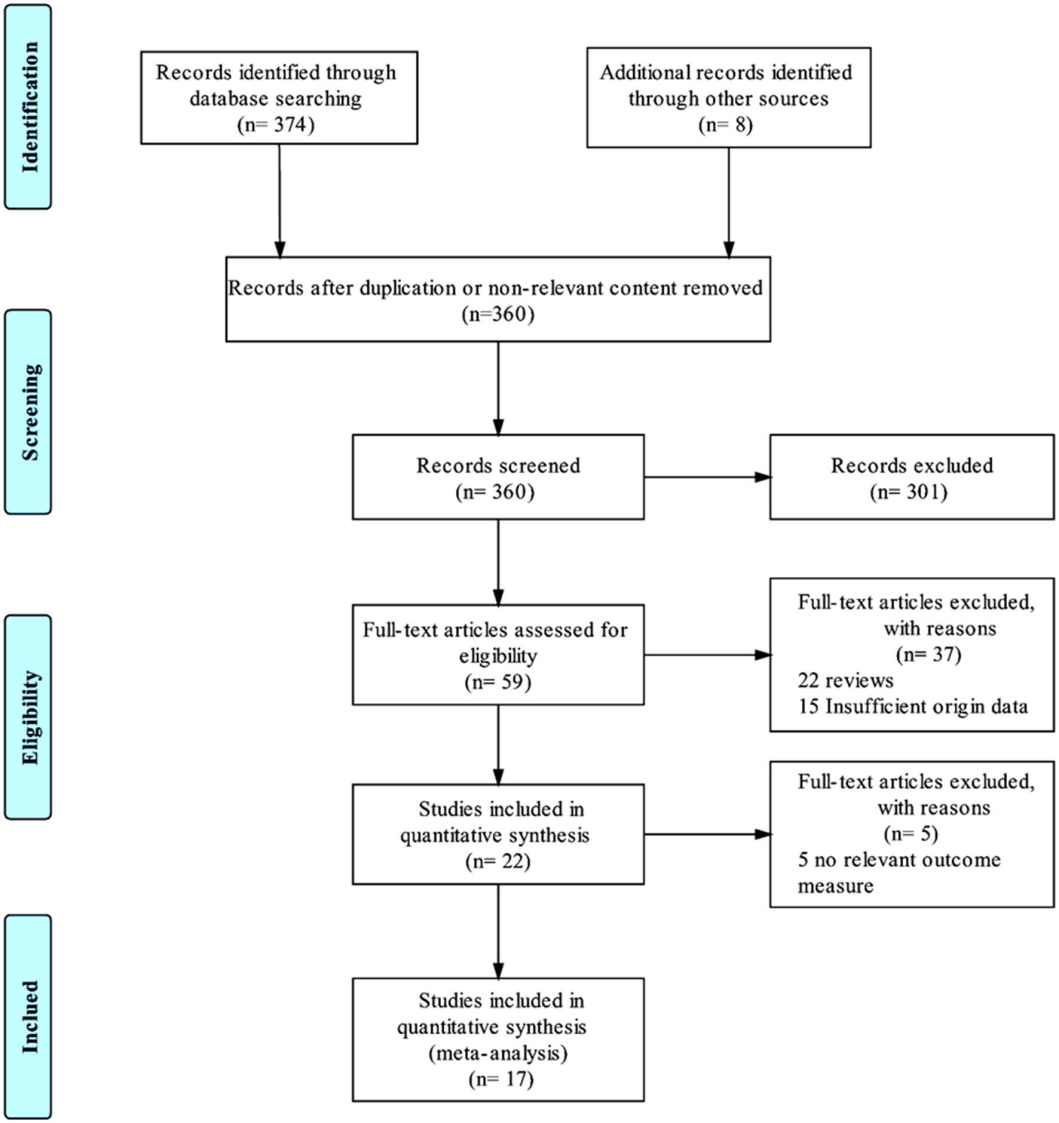
PRISMA flowchart. This flowchart represents the literature selection and elimination process taken to obtain the final 17 studies that were included in this meta-analysis.

During the systematic searching based on the searching strategies, 374 references from the electronic databases and eight additional records from other resources were identified and the last searching was performed on January, 30, 2019. After screening stage where the titles and abstracts of the retrieved records were reviewed, 360 records were excluded due to duplication or non-relevant content (Figure 1). Of the remaining 22 references, we excluded five (Jiménez et al., 2013; Mosley et al., 2015; Richardson et al., 2015; Narang et al., 2016; Bergfeld et al., 2017), because the primary outcomes presented by these trials did not meet the inclusion criteria. Thus, 17 studies (Mayberg et al., 2005; Lozano et al., 2008, 2012; Malone et al., 2009; Bewernick et al., 2010; Nahas et al., 2010; Kennedy et al., 2011; Holtzheimer et al., 2012; Puigdemont et al., 2012, 2015; Merkl et al., 2013; Schlaepfer et al., 2013; Dougherty et al., 2015; Accolla et al., 2016; Bergfeld et al., 2016; Fenoy et al., 2016; Williams et al., 2016), including eight studies target SCG (Mayberg et al., 2005; Lozano et al., 2008, 2012; Kennedy et al., 2011; Holtzheimer et al., 2012; Puigdemont et al., 2012, 2015; Merkl et al., 2013), 2 VC/VS (Malone et al., 2009; Dougherty et al., 2015), 2 EpC (Nahas et al., 2010; Williams et al., 2016), 1 Nac (Bewernick et al., 2010), 2 sMFB (Schlaepfer et al., 2013; Fenoy et al., 2016), 1 posterior gyrus rectus (Accolla et al., 2016), and 1 study targets vALIC (Bergfeld et al., 2016) were retrieved and included in this study. The study sample size was 233. All the studies reported demographic, clinical characteristics and surgical information, which are detailed in Table 1. The age of the sample population ranged from 42.0 to 50.7 years old. The follow-up period ranged from 3 months to 5 years. All the studies used the HDRS or MADRS as the primary outcome.

**Table 1:** Study characteristics.

**Study**	**Type**	**Demographics**				**Target**	**Stimulation parameters**			
		**Size-Diagnosis**	**Age (year)** **mean (SD)**	**Duration** ** (month)**	**Outcomes**		**Contact**	**Amplitude (V)**	**Frequency (Hz)**	**Pulse width (μs)**
Mayberg et al. (2005), Torres (2008)	Open label	6 MDD	46 (8.0)	6	HDRS; MADRS; AEs	SCG	Monopolar/bipolar	4.0	130	60
Lozano et al. (2008), Taghva et al. (2013)	Open label	20 MDD	47.4 (10.4)	12	HDRS; AEs	SCG	Monopolar	3.5–5.0	130	90
Kennedy et al. (2011), Williams et al. (2016)	Open label	20 TRD	47.4 (10.4)	36	HDRS; AEs	SCG	Monopolar	4.3	124.7	70.6
Lozano et al. (2008), Holtzheimer et al. (2012)	Open label	10 MDD 7 BPAD	42.0 (8.9)	24	HDRS; AEs	SCG	Monopolar	-	130	91
Malone et al. (2009), Lozano et al. (2012)	Open label	21 MDD	47.3 (6.1)	12	HDRS; MARDS; AEs	SCG	-	-	128.1	93.9
Bewernick et al. (2010), Puigdemont et al. (2012)	Open label	8 TRD	47.4 (11.3)	12	HDRS; MADRS; AEs	SCG	Bipolar	4.2	135	174.4
Nahas et al. (2010), Merkl et al. (2013)	Open label	6 MDD	50.7 (9.2)	3	HDRS; MADRS; AEs	SCG	Monopolar	5.0	130	90
Mayberg et al. (2005), Puigdemont et al. (2015)	RCT	5 Active5 Control	47.2 (12.23)	3	HDRS; MADRS; AEs	SCG	-	3.5–5.0	130–135	120–240
Malone et al. (2009), Puigdemont et al. (2015)	Open label	14 MDD 1 BPAD	46.3 (10.8)	6	HDRS; MADRS; AEs	VC/VS	-	6.7	127	113
Sartorius et al. (2010), Dougherty et al. (2015)	RCT	16 Active14 Control	47.7 (24.2)	4	MADRS; AEs	VC/VS	Monopolar/bipolar	<8	-	90, 210
	Open label	30 MDD	47.7 (24.2)	8	MADRS; AEs		Monopolar/bipolar	<8	-	90, 210
Nahas et al. (2010), Fenoy et al. (2016)	Open label	3 MDD 2 BPAD	44.4 (9.7)	7	HDRS; MADRS; AEs	EpC	-	2–4	60	-
Dougherty et al. (2015), Williams et al. (2016)	Open label	3 MDD 2 BPAD	44.4 (9.7)	60	HDRS; MADRS; AES	EpC	Double bipolar	4.5–6.5	130	90, 210
Bewernick et al. (2010), Accolla et al. (2016)	Open label	10 TRD	48.6 (11.7)	12	HDRS	Nac	Double negative	1.5–10.0	100–150	60-210
Jiménez et al. (2005), Schlaepfer et al. (2013)	Open label	6 MDD 1 BPAD	42.6 (9.8)	3	HDRS; MADRS; AEs	sMFB	Bipolar	2–3	130	60
Schlaepfer et al. (2013), Fenoy et al. (2016)	Open label	4 MDD	46.3 (8.9)	6.5	HDRS; MADRS; AEs	sMFB	Double negative	3.2	130	60
Merkl et al. (2013), Accolla et al. (2016)	Open label	5 MDD	45.2 (12.89)	3	HDRS;	PGR	Monopolar	5	130	90
Kuo et al. (2014), Bergfeld et al. (2016)	Open label	25 TRD	53.2 (8.4)	12	HDRS; MADRS; AEs	vALIC	-	2.5–6.0	130–180	90
	RCT	9 Active 7 Control	-	-	HDRS; MADRS; AEs		-	2.5–6.0	130–180	90

*RCT, Randomized Controlled Trials; MDD, major depressive disorder; BPAD, bipolar affective depression; TRD, treatment-resistant depression; HRSD, Hamilton Rating Scale for Depression; MADRS, Montgomery-Asberg Depression Rating Scale; AEs, Adverse Events; SCG, Subcallosal Cingulate Gyrus; VC/VS, Ventral Capsule/Ventral Striatum; NAc, Nucleus Accumbens; EpC, Epidural prefrontal Cortical; vALIC, Ventral Anterior Limb of the Interna Capsule; sMFB, Supero-lateral branch of the Medial Forebrain Bundle; PGR, Posterior Gyrus Rectus; -, Not Available; data presented with mean (SD)*.

### Evaluation of the Response Rate in the Open-Label Studies and RTCs

**Figure 2:**
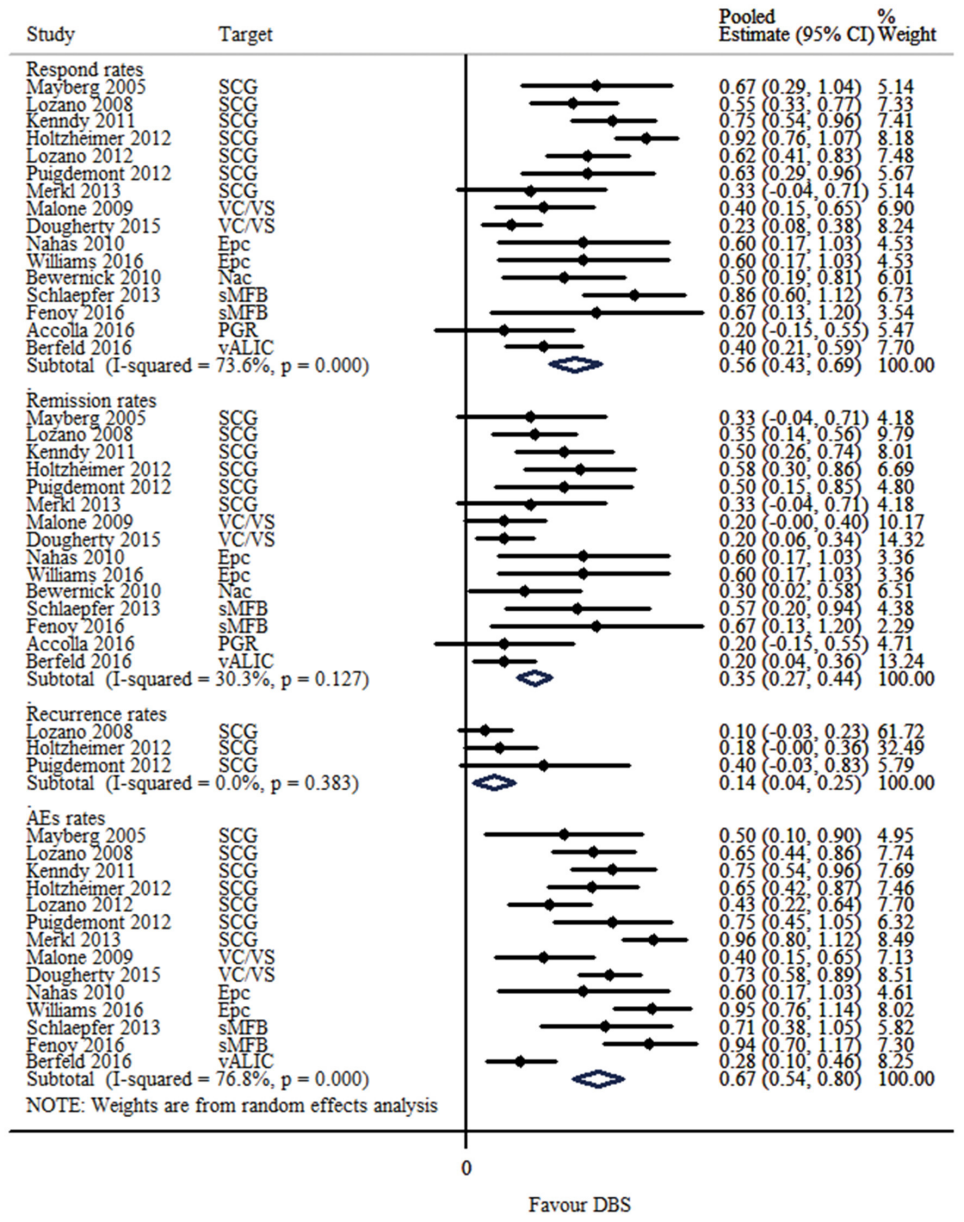
Forest plots showing a summary of evaluation of respond, remission, recurrence and AEs rates in the open-label studies.

**Figure 3:**
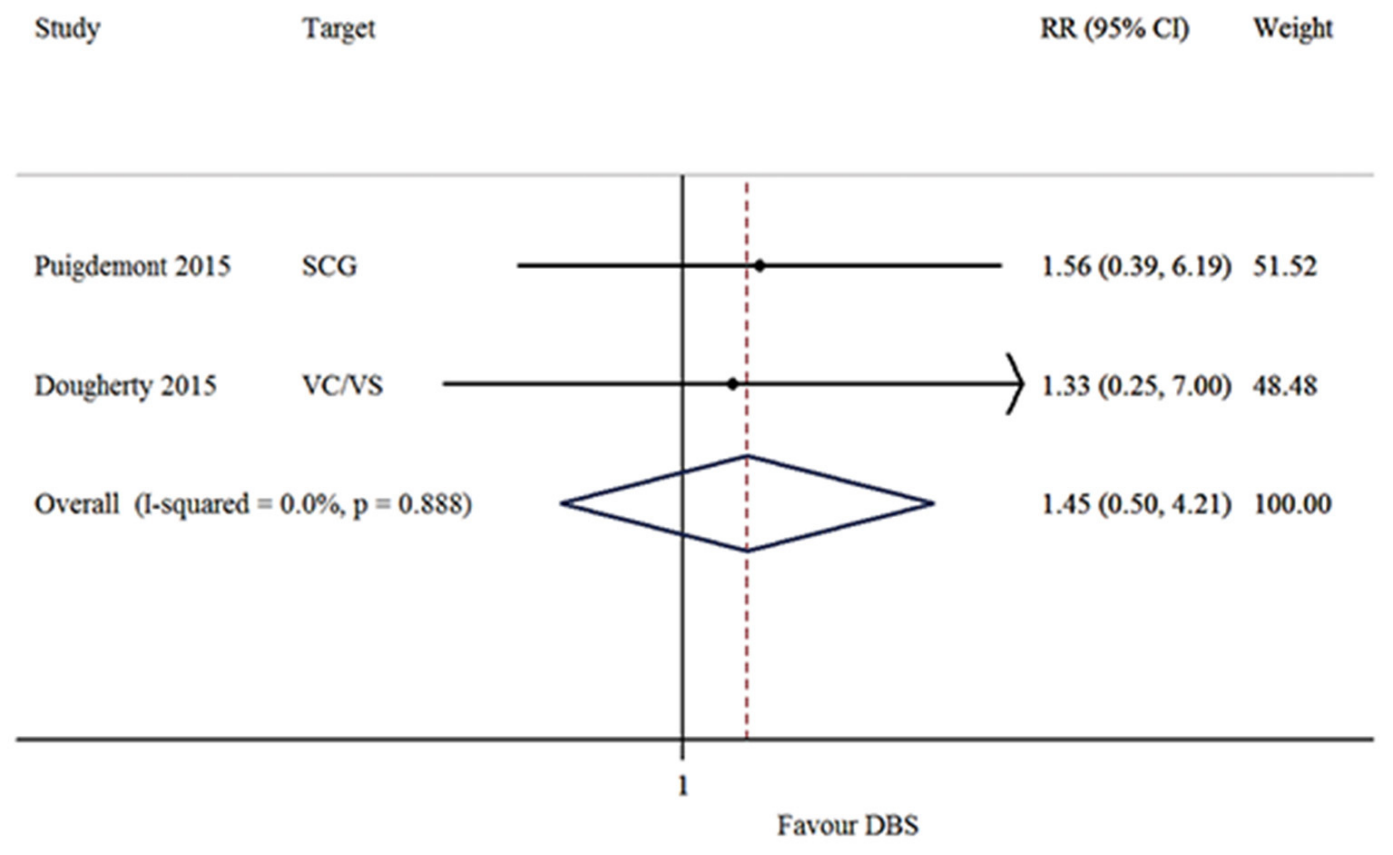
Forest plots showing a summary of the evaluation of the respond rates in the RCTs.

The response, remission and recurrence rate were used to evaluate the efficacy and the AEs rate was used to investigate the safety of the DBS treatment for TRD. Table 2 summarizes these finding from the open-label studies, and Table 3 represents the included RCTs. At total of 16 open-label studies (Mayberg et al., 2005; Lozano et al., 2008, 2012; Malone et al., 2009; Bewernick et al., 2010; Nahas et al., 2010; Kennedy et al., 2011; Holtzheimer et al., 2012; Puigdemont et al., 2012; Merkl et al., 2013; Schlaepfer et al., 2013; Dougherty et al., 2015; Accolla et al., 2016; Bergfeld et al., 2016; Fenoy et al., 2016; Williams et al., 2016) and 2 RCTs (Dougherty et al., 2015; Puigdemont et al., 2015) compared the response rate and showed a statistically significant heterogeneity (*I*^2^ = 73.6%, *p* < 0.0001^*^), using a random model (Figure 2). According to the result of the meta-analyses, the pooled estimate was 0.56 (95% CI = 0.43–0.69) in the non-RCT group (Figure 2) and the pooled response-rate value was 1.45 (95% CI = 0.50–4.21) in only two RCTs (Figure 3).

**Table 2:** Summary of the efficacy and safety assessments (open-label studies).

**Study**	**Target**	**Efficacy assessments**			**Safety assessments**		
		**Response rate**	**Remission rate**	**Recurrence rate**	**AEs**	**Suicide**	**Suicidal attempt**
Mayberg et al. (2005), Torres (2008)	SCG	66.7%	33.3%	-	50.0%	-	-
Lozano et al. (2008), Taghva et al. (2013)	SCG	55.0%	35.0%	10.0%	65.0%	-	-
Kennedy et al. (2011), Williams et al. (2016)	SCG	75.0%	50.0%	-	75.0%	2	2
Lozano et al. (2008), Holtzheimer et al. (2012)	SCG	91.7%	58.3%	17.6%	64.7%	-	4
Malone et al. (2009), Lozano et al. (2012)	SCG	61.9%	-	-	42.9%	1	1
Bewernick et al. (2010), Puigdemont et al. (2012)	SCG	62.5%	50.0%	40.0%	75.0%	-	1
Nahas et al. (2010), Merkl et al. (2013)	SCG	33.3%	33.3%	-	100.0%	-	-
Malone et al. (2009), Puigdemont et al. (2015)	VC/VS	40.0%	20.0%	-	40.0%	-	4
Sartorius et al. (2010), Dougherty et al. (2015)	VC/VS	23.3%	20.0%	-	73.3%	1	5
Nahas et al. (2010), Fenoy et al. (2016)	EpC	60.0%	60.0%	-	60.0%	-	-
Dougherty et al. (2015), Williams et al. (2016)	EpC	60.0%	60.0%	-	100.0%	-	4
Bewernick et al. (2010), Accolla et al. (2016)	Nac	50.0%	30.0%	-	-	1	1
Jiménez et al. (2005), Schlaepfer et al. (2013)	sMFB	85.7%	57.1%	-	71.4%	-	-
Schlaepfer et al. (2013), Fenoy et al. (2016)	sMFB	66.7%	66.7%	-	100.0%	-	-
Merkl et al. (2013), Accolla et al. (2016)	PGR	20.0%	20.0%	-	-	-	-
Kuo et al. (2014), Bergfeld et al. (2016)	vALIC	40.0%	20.0%	-	28.0%	1	6

*AEs, Adverse Events; SCG, Subcallosal Cingulate Gyrus; VC/VS, Ventral Capsule/Ventral Striatum; NAc, Nucleus Accumbens; EpC, Epidural prefrontal Cortical; vALIC, Ventral Anterior Limb of the Interna Capsule; sMFB, Supero-lateral branch of the Medial Forebrain Bundle; PGR, Posterior Gyrus Rectus; -, Not Available*.

**Table 3:** Summary of the efficacy and safety assessments (randomized controlled studies).

**Study**	**Target**	**Efficacy assessments**			**Safety assessments**		
		**Response rate**	**Remission rate**	**Recurrence rate**	**AEs**	**Suicide**	**Suicidal attempt**
Puigdemont et al. (2015), Mayberg et al. (2005)	SCG	Active: 80%Control: 40%	Active: 80% Control: 40%	Active: 0%Control: 40%	-	-	-
Sartorius et al. (2010), Dougherty et al. (2015)	VC/VS	Active: 20%Control: 14.2%	-	-	Active: 66.7% Control: 26.7%	-	2
Kuo et al. (2014), Bergfeld et al. (2016)	vALIC	-	-	-	-	1	6

*AEs, Adverse Events; SCG, Subcallosal Cingulate Gyrus; VC/VS, Ventral Capsule/Ventral Striatum; vALIC, Ventral Anterior Limb of the Interna Capsule; -, Not Available*.

#### Evaluation of the Remission Rate in the Open-Label Studies

At total of 15 open-label studies (Mayberg et al., 2005; Lozano et al., 2008; Malone et al., 2009; Bewernick et al., 2010; Nahas et al., 2010; Kennedy et al., 2011; Holtzheimer et al., 2012; Puigdemont et al., 2012; Merkl et al., 2013; Schlaepfer et al., 2013; Dougherty et al., 2015; Accolla et al., 2016; Bergfeld et al., 2016; Fenoy et al., 2016; Williams et al., 2016) compared the remission rate with no statistical heterogeneity (*I*^2^ = 30.3%, *p* = 0.127), using a fixed model. According to the results of the meta-analyses, the pooled estimate was 0.32 (95% CI = 0.25–0.39) (Figure 2).

#### Evaluation of the Recurrence Rate in the Open-Label Studies

Three open label studies (Lozano et al., 2008; Holtzheimer et al., 2012; Puigdemont et al., 2012) compared the recurrence rate with no statistical heterogeneity (*I*^2^ = 0.0%, *p* = 0.383), using fixed model. According to the results of the meta-analyses, the pooled estimate was 0.14 (95% CI = 0.04–0.25) (Figure 2).

### Evaluation of the AEs Rate in the Open-Label Studies

At total of 14 open-label studies (Mayberg et al., 2005; Lozano et al., 2008, 2012; Malone et al., 2009; Nahas et al., 2010; Kennedy et al., 2011; Holtzheimer et al., 2012; Puigdemont et al., 2012; Merkl et al., 2013; Schlaepfer et al., 2013; Dougherty et al., 2015; Bergfeld et al., 2016; Fenoy et al., 2016; Williams et al., 2016) compared the AEs rate and showed a statistically significant heterogeneity (*I*^2^ = 76.8%, *p* < 0.0001^*^), using a random model. According to the results of the meta-analyses, the pooled estimate was 0.67 (95% CI = 0.54–0.80) (Figure 2). Of the included articles, nine reported the rate of suicide and suicidal attempt. The median rate of suicidal attempt was 16.7% (ranged from 4 to 80%) and of suicide was 4.8% (ranged from 3.3 to 12.5%).

### Heterogeneity Analyses

**Figure 4:**
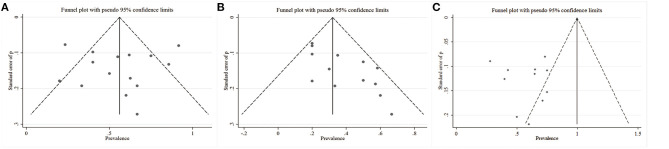
Funnel plots with the pseudo 95% confidence limits. **(A)** Response rates; **(B)** Remission rates; **(C)** Adverse events rates.

**Figure 5:**
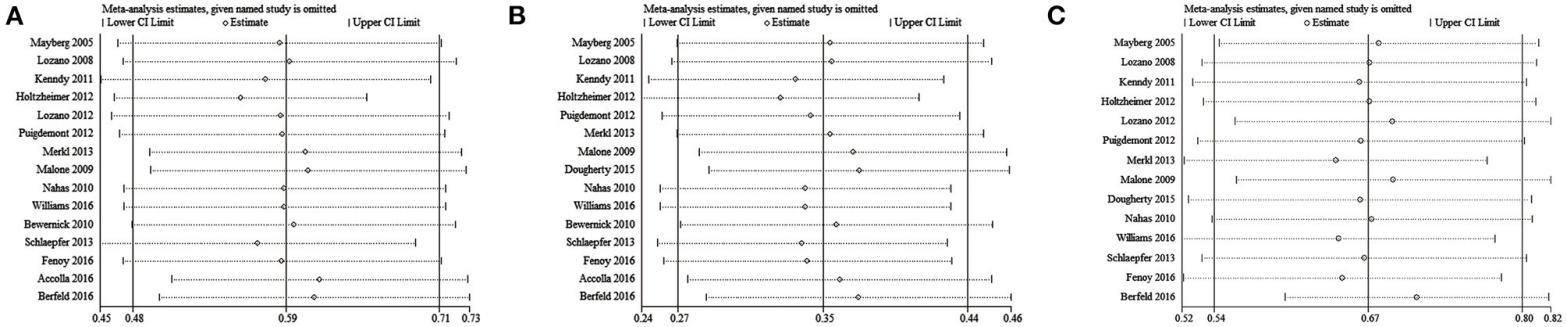
Sensitivity analysis. **(A)** Response rates; **(B)** Remission rates; **(C)** Adverse events rates.

To assess the statistical heterogeneity for response, remission and AEs rate, funnel plots (Figures 4A–C) were generated and a sensitivity analysis (Figures 5A–C) was performed to evaluate the publication bias and sources of heterogeneity. In the final analysis, by omitting one study at a time, none of the studies attributed to the heterogeneity in the response rate, while three studies contributed to the heterogeneity in the AEs rate (Merkl et al., 2013; Fenoy et al., 2016; Williams et al., 2016). Moreover, as shown in the Table 4, the studied outcomes of the meta-analyses were reliable, since the effect of a larger or smaller sample size was excluded. When we exclude the larger or smaller sample size studies, they did not influence the pooled outcomes or the heterogeneity.

**Table 4:** Results of the sensitivity analysis (open-label studies).

**Outcome**	**Random model**			**Omitted large sample study**			**Omitted small sample study**		
	**PE**	**95% CI**	***P*-value**	**PE**	**95% CI**	***P*-value**	**PE**	**95% CI**	***P*-value**
Respond rate	0.56	0.43, 0.69	<0.0001^*^	0.59	0.48, 0.71	0.001^*^	0.55	0.42,0.69	<0.0001^*^
Remission rate	0.35	0.27, 0.44	0.127	0.37	0.28, 0.46	0.215	0.36	0.27. 0.45	0.225
Recurrence rate	0.14	0.04, 0.25	0.383	0.21	0.04, 0.38	0.347	0.13	0.02, 0.23	0.503
AEs rate	0.89	0.85, 0.93	<0.0001^*^	0.90	0.86, 0.94	<0.0001^*^	0.81	0.76, 0.87	<0.0001^*^

PE, Pooled Estimate; 95% CI, 95% confidence intervals;

* *significant difference*.

### Risk of Bias Across Studies

We reduced the bias in our study as much as possible. We used reasonable search strategies, and the contribution of three independent authors contributed to reducing the sampling bias. In addition, we used explicit and rigid inclusion criteria, independent searching and a sensitivity analysis to contribute to the reduction of selection bias. Moreover, no funding source influenced the reporting bias. However, almost all the included studies were open-label studies, which will inherently result in bias indicated by an asymmetry in funnel plots.

## Discussion

### Summary of Evidence

Currently, DBS therapy is still in the stage of exploration and cannot be used as a recommended intervention. Most patients with TRD, who undergo DBS treatment, also receive antidepressant treatment, psychotherapy and other neuromodulation therapies, according to the 2016 guidelines by the Canada Network for Mood and Anxiety Treatment (CANMAT) for management of major depressive disorder (MDD) in adults (Kennedy et al., 2016). Previous studies in this field are largely non-randomized controlled small-sample studies. In the scope of reviewing the published literature, the SCG was the main target region in the brain used for depression. Other regions were also published, including the EpC, the sMFB, the posterior gyrus rectus, the vALIC, the VC/VS, and the NAc. Case reports mention a potential region of the inferior thalamic peduncle conveying thalamo-cortical information (Jiménez et al., 2005) and habenula associating with monoaminergic neurotransmission (Sartorius et al., 2010). A preliminary trial of DBS described that targeting the nucleus accumbens was more promising than the caudate, as evaluated by positron emission computed tomography (PET) (Millet et al., 2014). An exploratory meta-analysis was published in 2014 (Smith, 2014), which concluded that the procedure may be 71% more effective than a sham treatment, and one out of three patients with depression were expected to benefit from DBS. However, the estimates of the sham response were by expert opinions plus a random number software rather than practice data. Mosley et al. (2015) conducted a systematic review to exam the impact of DBS on depression. They did not make a concrete conclusion and called for methodological refinements.

In our study, DBS, for all the discussed targets, seemed to be a potential treatment option for TRD. On the one hand, the response and remission rates were respectively 56 and 32%, while the recurrence rate was relatively low (14%), suggesting that this treatment might be considered as promising. On the other hand, the AEs rate was common (67%) and moderate, which can be resolved by the corresponding therapy. However, suicide and suicidal ideation still occurred in the patients who received this treatment. Two studies (Dougherty et al., 2015; Puigdemont et al., 2015) improved the design to perform RCTs and found that there were no significant differences in the active and sham groups. This controversial opinion should be studied more extensively, with more blinded, randomized controlled trials. Among the included studies, eight studies (Mayberg et al., 2005; Malone et al., 2009; Merkl et al., 2013; Schlaepfer et al., 2013; Dougherty et al., 2015; Accolla et al., 2016; Fenoy et al., 2016) referred the short-term (<1 year) effect in DBS for TRD, and the respond rates ranged from 20 to 91.7% and the remission rates from 20 to 66.7%. In addition, eight studies (Lozano et al., 2008, 2012; Bewernick et al., 2010; Kennedy et al., 2011; Holtzheimer et al., 2012; Puigdemont et al., 2012; Bergfeld et al., 2016; Williams et al., 2016) assessed the long-term (more than 1 year) effect, and the respond rates ranged from 40 to 75% and the remission rates were from 20 to 60%. Specifically, in a 5-year follow-up study (Williams et al., 2016), the mean improvements from the pre-implant baseline for the HRSD were 41.2, 53.8, and 45% in 1 year, 2 years, and 5 years, respectively. In addition, 3 of 5 (60%) patients continued to be in remission at the end of the follow up. The evaluation of the side effects of DBS for TRD requires a consideration of various factors, including the surgical procedures, infection and the stimulation in unrelated brain regions. No evidence currently reveals that the approach damages neurocognitive performance. However, DBS treatment could be conducive to psychiatric disorders, manic episodes and other psychiatric disorders, but these conditions are transient and can be reversed by adjusting the parameters (Fitzgerald, 2008).

The exact mechanism of DBS is unknown and studies are ongoing on this regard. Some studies have demonstrated that the gamma oscillations inhibition and the facilitation of theta-gamma coupling by DBS is probably mediated by activation of inhibitory circuits in SCG and the enhancement of plasticity in the frontal cortex (Sun et al., 2015). Depression is generally considered to involve three compartments of the neurocircuitry including the dorsal, ventral, and modulatory. The dorsal compartment mediates the cognitive and motor aspects, and the ventral compartment is associated with the somatic and vegetative aspects. The modulatory system mediates the mutual interactions of these two compartments through an inhibitory pathway consisting of the amygdala, hippocampus, rostral cingulate cortex, and the hypothalamic-pituitary-adrenal axis (Morishita et al., 2014).

During the last decade, several studies have utilized anatomical and functional neuroimaging modalities to investigate the neurobiology of depression (Holtzheimer and Mayberg, 2010). Structural neuroimaging studies consistently report smaller medial volumes, primarily the hippocampus, amygdala, and the entorhinal cortex, in depressive patients (Sheline et al., 1996, 1998; Hastings et al., 2004; Videbech and Ravnkilde, 2004). Zhang et al. (2009) suggested that the subtle structural/functional dysfunction in the right anterior cingulate, insula, caudate tail and amygdala-parahippocampal regions was associated with depression. Moreover, it is reported that non-responsive patients have a more organized white matter (Taylor et al., 2008) and a smaller right medial frontal and striatal volumes compared to responsive patients and comparison subjects (Shah et al., 2002). Functional imaging studies show that depressive patients have a higher ratio of amygdalar-hippocampal to cortical blood flow (Hornig et al., 1997) and lower concentrations of occipital γ-aminobutyric acid (Price et al., 2009). The aim of the future studies should involve defining the best localization within that target area. Moreover, imaging studies are important to elucidate the mode of action.

### Limitations

There are several limitations in this study. The specificity of the DBS surgery and the relevant ethical issue attributes to the difficulty of performing high quality and large sample RCTs. We, therefore, performed this meta-analysis using the rates to obtain a more reliable conclusion by pooling rates from multiple studies. In addition, the study implementation, patient characteristics, and different sample sizes also contribute to the bias. However, we illustrated the clinical heterogeneity by performing a sensitivity analysis. Further studies should refine the methodology, investigate the optimal targets structures and stimulation parameters, and maximize the consistency of outcomes measurement to make comparisons feasible. Moreover, it is of extreme importance to analyze the exact network effects to understand the neurobiological mechanisms.

### Conclusions

We concluded that the DBS treatment had good prospects. However, the problem of the reported adverse events is worth paying attention to and resolving, because it could lower the quality of life. Further basic scientific research is needed to search for more optimal brain structures to modulate the neural circuits and to investigate the mechanisms of underlying the DBS treatment.

## Data Availability Statement

The raw data supporting the conclusions of this article will be made available by the authors, without undue reservation.

## Author Contributions

KZ and XX designed the topic and reviewed the manuscript. LS, JZ, WH, CZ, YW, CL, BZ, and XW collected the references and did the statistical analysis. JM and YLW wrote the manuscript. All authors contributed to the article and approved the submitted version.

## Conflict of Interest

The authors declare that the research was conducted in the absence of any commercial or financial relationships that could be construed as a potential conflict of interest.
